# School Absenteeism As an Adjunct Surveillance Indicator: Experience during the Second Wave of the 2009 H1N1 Pandemic in Quebec, Canada

**DOI:** 10.1371/journal.pone.0034084

**Published:** 2012-03-30

**Authors:** Christelle Aïcha Kom Mogto, Gaston De Serres, Monique Douville Fradet, Germain Lebel, Steve Toutant, Rodica Gilca, Manale Ouakki, Naveed Zafar Janjua, Danuta M. Skowronski

**Affiliations:** 1 Department of Social and Preventive Medicine, Université Laval, Quebec City, Quebec, Canada; 2 Institut national de santé publique du Québec, Quebec City, Quebec, Canada; 3 British Columbia Centre for Disease Control, Vancouver, British Columbia, Canada; 4 School of Population and Public Health, University of British Columbia, Vancouver, British Columbia, Canada; National Institutes of Health, United States of America

## Abstract

**Background:**

A school absenteeism surveillance system was implemented in the province of Quebec, Canada during the second wave of the 2009 H1N1pandemic. This paper compares this surveillance approach with other available indicators.

**Method:**

All (3432) elementary and high schools from Quebec were included. Each school was required to report through a web-based system any day where the proportion of students absent for influenza-like illness (ILI) exceeded 10% of current school enrolment.

**Results:**

Between October 18 and December 12 2009, 35.6% of all schools met the 10% absenteeism threshold. This proportion was greater in elementary compared to high schools (40% vs 19%) and in smaller compared to larger schools (44% vs 22%). The maximum absenteeism rate was reached the first day of reporting or within the next two days in 55% and 31% of schools respectively. The first reports and subsequent peak in school absenteeism provincially preceded the peak in paediatric hospitalization by two and one weeks, respectively. Trends in school surveillance otherwise mirrored other indicators.

**Conclusion:**

During a pandemic, school outbreak surveillance based on a 10% threshold appears insufficient to trigger timely intervention within a given affected school. However, school surveillance appears well-correlated and slightly anticipatory compared to other population indicators. As such, school absenteeism warrants further evaluation as an adjunct surveillance indicator whose overall utility will depend upon specified objectives, and other existing capacity for monitoring and response.

## Introduction

Influenza causes significant morbidity and mortality in elderly people but school children play an important role in virus transmission [1], [2]. They are often affected early during outbreaks and because of their high contact rates, are thought to amplify and accelerate spread in the general population [3], [4]. For this reason, surveillance of school outbreaks through absenteeism tracking has been used by public health authorities for the purpose of early monitoring of increase in community-level influenza activity. Few studies, however, have evaluated school-based surveillance [1], [3], [5], [6] and fewer have assessed the parameters for reporting (eg. threshold of absenteeism or duration of reporting) for optimal surveillance [1], [7].

In the context of a pandemic, the relevance of school absenteeism surveillance may be greater. Guidelines for school closure during an outbreak have been developed in order to slow or mitigate epidemic intensity by disrupting the branching chains of transmission through the contact networks of school children. Efficient and accurate school absenteeism surveillance is required for school closures to be timely and effective [4]–[13]. Several studies have evaluated the possible epidemiological impact of school closure and the associated costs [8]–[15]. They suggest that well-designed absenteeism surveillance programs are needed to minimize societal disruption while maximizing benefits through this intervention [7].

Some provinces of Canada rely on school absenteeism as a surveillance indicator for community influenza activity [16], but no study has yet been published on the conditions to optimize its usefulness. During the fall of 2009, a school absenteeism surveillance system was implemented in the province of Quebec to better assess the impact of the second wave of pandemic influenza A (H1N1) in school-age children. This paper describes the characteristics and the evolution of school absenteeism during the fall of 2009, and compares the results of school-based surveillance to other influenza indicators.

## Methods

### Ethics Statement

This study was based on surveillance data from aggregated administrative results for each school. For this reason, ethics board review and individual consent were not required.

### Surveillance of school absenteeism

Surveillance for school absenteeism related to influenza-like illness (ILI) was undertaken by all (3432) elementary and high schools (private or public) in the province of Quebec, Canada. Each school was required by the Ministère de l'Éducation, du Loisir et du Sport (MELS) to immediately report through a web-based system any day where the proportion of students absent for an ILI exceeded 10% of the current school enrolment [17]. The choice of the 10% threshold was based on criteria specified by the Public Health Agency of Canada for influenza outbreaks [17]. ILI was defined as a respiratory disease associated with fever and cough and one or more of sore throat, arthralgia, myalgia, or prostration. Schools were requested to continue reporting the rate of absenteeism for seven days following the last day meeting 10%. Data were integrated in a geographical information system. Data collection started on the US CDC week 42 (18^th^ of October 2009), and ended on week 49 (12^th^ of December 2009).

### Other surveillance systems for influenza

Provincial surveillance for laboratory-confirmed cases and hospitalizations due to pandemic influenza was conducted through a network of accredited laboratories all performing polymerase chain reaction (PCR) testing according to guidelines of the National Microbiology Laboratory. Laboratories entered data for each positive case into a web-based provincial database on a real-time-of-result basis. Reporting to regional public health units of laboratory-confirmed influenza hospitalizations was mandatory and compiled on a date of admission basis. These data were analysed daily by the public health network.

### Statistical analysis

Although schools were instructed that the threshold for first reporting was a 10% daily absenteeism rate, some schools started reporting before reaching this threshold. For the analysis, schools were considered affected only from the first day the absenteeism rate was ≥10%. The weekly incidence was calculated by dividing the number of schools reaching the ≥10% daily absenteeism threshold for the first time (newly affected) in a particular week by the total number of schools at risk (not yet affected).
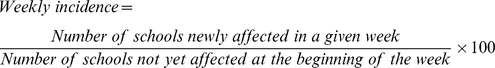



The weekly prevalence was calculated by dividing the number of schools reporting ≥10% absenteeism at least one school day during a particular week (whether or not it was the first time) by the total number of schools. Multiple days of ≥10% absenteeism within a given school for a given week were counted only once.
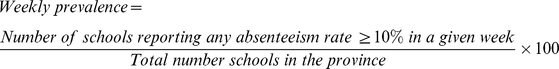
The cumulative incidence of affected schools was calculated by dividing the number of schools that reached the ≥10% daily absenteeism threshold at least once between the beginning of the study period and the week of interest (total affected), by the total number of schools in the province.
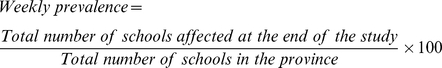



The weekly incidence of reporting schools in different regions was plotted in a geographical information system. The geographic information system was constructed using free Open Source software and frameworks: MapServer, PostgreSQL/PostGis, JavaScript OpenLayers, and ExtJS/GeoExt.

Results from school surveillance were compared to those of surveillance based on the weekly distribution of all influenza PCR positives tests and weekly distribution of all PCR-confirmed hospitalizations in the general population and in the population of children aged 5 to 17 years by week of hospital admission. A positive specimen counted for the week during which it was collected. To quantify the similarity between the epidemic curves for each surveillance indicator, we calculated and tested the correlation by using the non-parametric Spearman coefficient.

The Chi-square test was used to compare proportions, whereas comparison of medians was done with the Kruskal-Wallis non-parametric test. Linear regression was used to assess the effect of school size and education level (elementary vs high schools) of the maximum absenteeism rates and duration of reporting.

## Results

Among the 3432 schools in the province, there were 2477 (72.2%) elementary schools, 736 (21.4%) high schools and 218 (6.4%) schools with both elementary and high school levels (E+H). The total enrolment in elementary schools during the study period was 527,234 students for a mean/median of 213/186 students per school, compared to 415,618 students in high schools with mean/median 565/465 students per school. E+H schools had 64,310 students and a mean/median size of 295/184 students per school.

A total of 1352 schools reported any absenteeism. Of these, 129 schools reported absenteeism below the 10% threshold and never reached this threshold whereas 1223 (35.6%) schools reported absenteeism ≥10%. Among these 1223 schools, 81% (n = 992) were elementary schools, 11.5% (n = 141) were high schools and 7.5% (n = 90) were E+H schools.

### Description of school absenteeism during the study period

**Figure 1:**
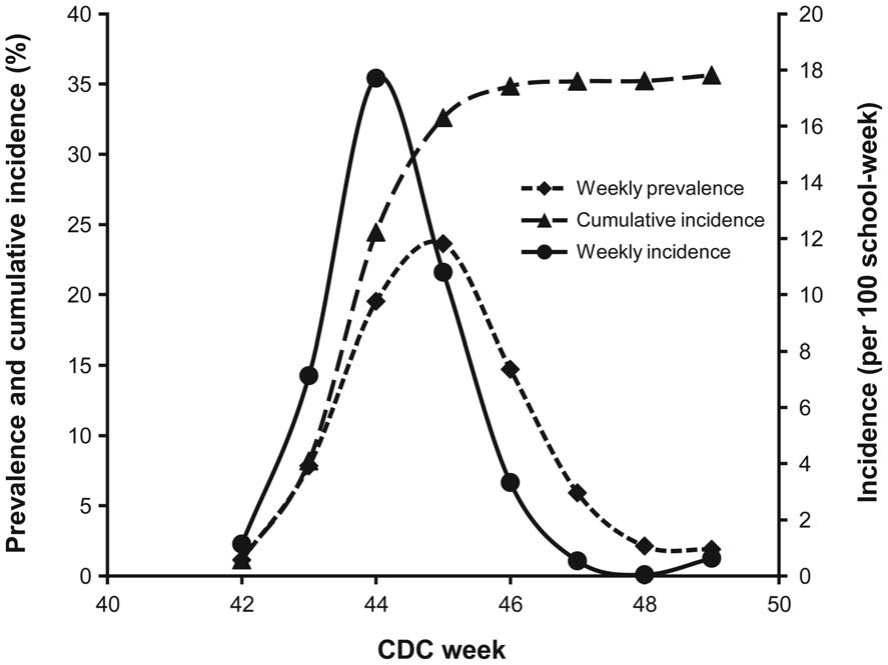
Weekly incidence, weekly prevalence and cumulative incidence of schools reporting an absenteeism rate ≥10%.

On the first week of surveillance (CDC week 42), 39 schools (1.1% of schools) reported an absenteeism rate of 10% or more. The maximum number of schools newly reporting absenteeism ≥10% for both elementary and high schools in a single week occurred two weeks later (week 44) when there were 558 newly affected schools for an incidence of 17.7 per 100 school-weeks (Figure 1). After that week, the incidence rate dropped rapidly and was only 0.62 per 100 school-weeks (14 schools newly affected) by week 49. The maximum weekly prevalence of schools reporting absenteeism ≥10% occurred at week 45 during which it reached 23.6% (811 schools) (Figure 1). For the entire follow-up period, the cumulative incidence of elementary schools reporting an absenteeism rate of ≥10% was higher compared to high schools (40% *vs* 19%, p = 0.01). This higher cumulative incidence in elementary schools was observed regardless of school size (Table 1). Schools with 100 to 299 students most often reached the ≥10% absenteeism threshold (44%) while this was less often reached (22%) among schools with ≥500 students (Table 1).

**Table 1:** Cumulative incidence (%) of schools that reported an absenteeism rate ≥10% by size and level.

School level	Total number of students in schools					
	Total%(n/N)	<100%(n/N)	100–299%(n/N)	300–499%(n/N)	≥500%(n/N)	P value
Elementary schools	40%(992/2478)	35%(257/733)	46%(481/1052)	39%(218/555)	26%(36/138)	<0.0001
High schools	19%(141/736)	7%(14/212)	39%(27/88)	31%(29/94)	21%(71/342)	<0.0001
Elementary+High (E+H) schools	41%(90/218)	66%(43/65)	41%(36/88)	17%(6/35)	17%(5/30)	<0.0001
All schools	36%(1223/3432)	31%(314/1010)	44%(544/1228)	37%(253/684)	22%(112/510)	<0.0001

**Figure 2:**
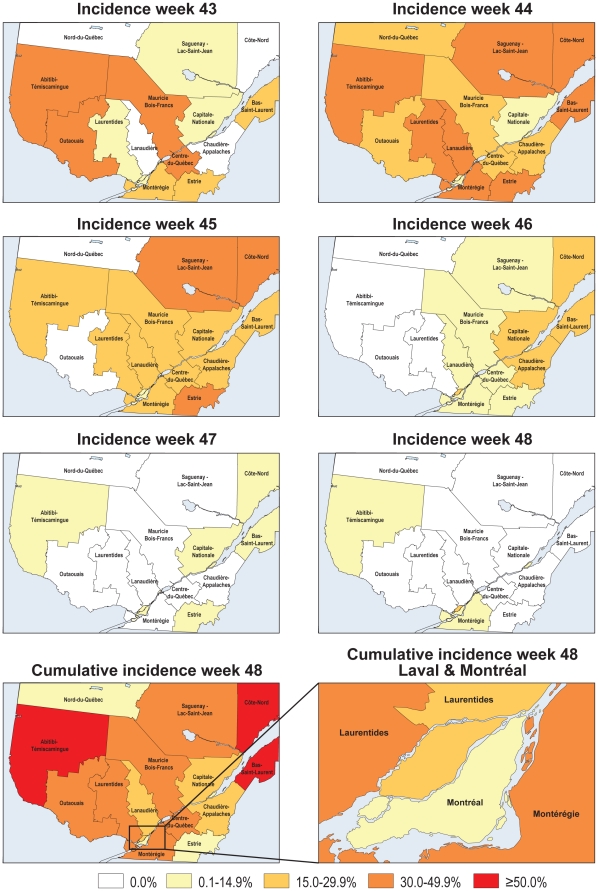
Weekly incidence of affected schools from week 43 to week 48(six upper panels); cumulative incidence of affected schools in the province at the end of the study (bottom left panel); cumulative incidence of affected schools in the regions of Montreal and Laval at the end of the study (bottom right panel).

The incidence of schools with absenteeism ≥10% per region changed over time, with western regions of the province being affected slightly earlier (week 43) in the epidemic than the rest of the province (Figure 2). The following week (week 44), similarly high incidence was observed in the other regions and corresponded to the peak incidence for all but one region. Montreal, which is the region with the highest population density in the province, had the lowest cumulative incidence of affected schools during the second wave of the pandemic (Figure 2 lower right panel).

**Figure 3:**
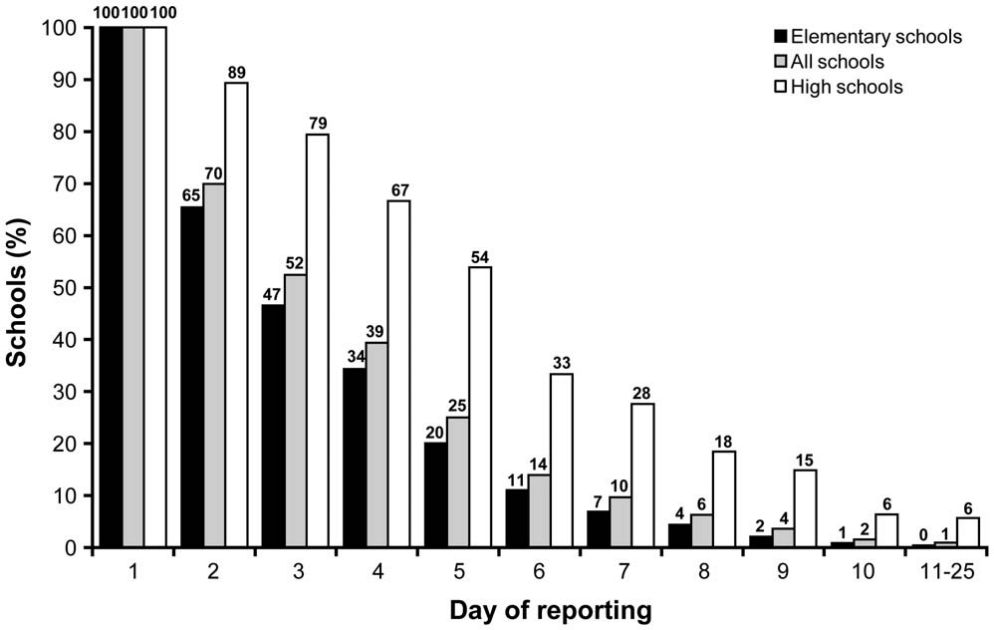
Proportion of elementary and high schools that continued to report an absenteeism rate ≥10% since the day of first reporting by school size.

**Figure 4:**
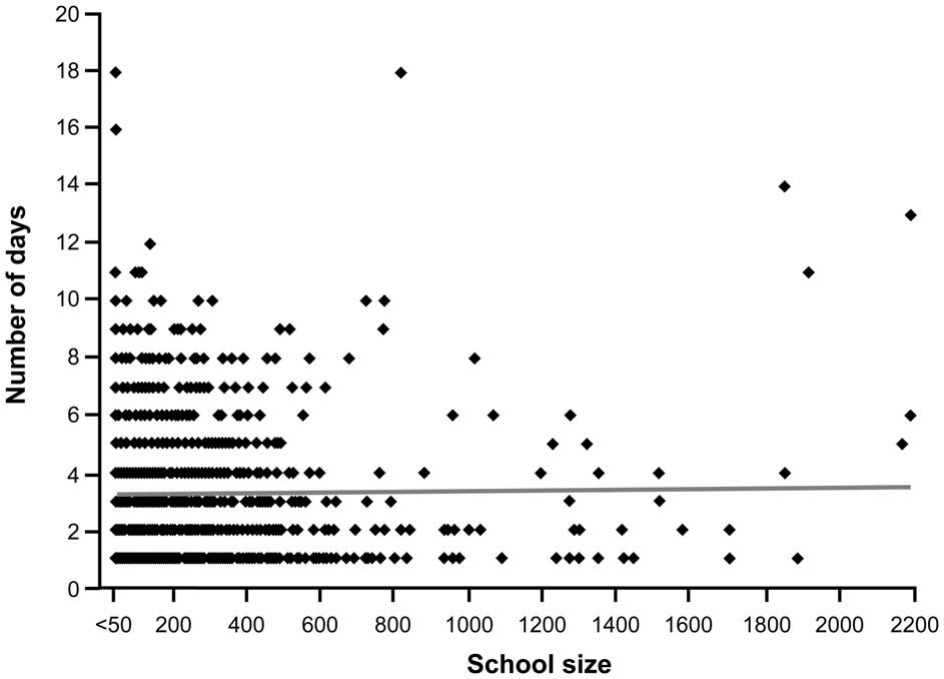
Distribution of the number of days of reporting by school size (all schools) and regression line.

The overall mean absenteeism rate on the first day of reporting was 15.3%±0.5% (n = 1223) for all schools and was similar in elementary and high schools (15.9% *vs* 15.2%). The overall mean absenteeism rates decreased to 12.7% (n = 1024) the 2^nd^ day (p<0.001), 10.9% (n = 904) the 3^rd^ day (p<0.001), and 8.5% (n = 800) the fourth day (p<0.001). Among the 141 high schools that reached the 10% absenteeism threshold, 67% were still above this threshold on the 4^th^ day of follow-up (Figure 3) compared to 34% of the 992 elementary schools (p = 0.01). Only 6% (9/141) of high schools and 1% (10/992) elementary schools still met the 10% threshold at 10 days of follow up (Figure 3). The number of days of reporting showed greater variability in small compared to larger schools. (Figure 4) However, in linear regression analysis, neither the school size (p = 0. 96) nor the education level (elementary vs high school) (p = 0.91) were significant.

**Figure 5:**
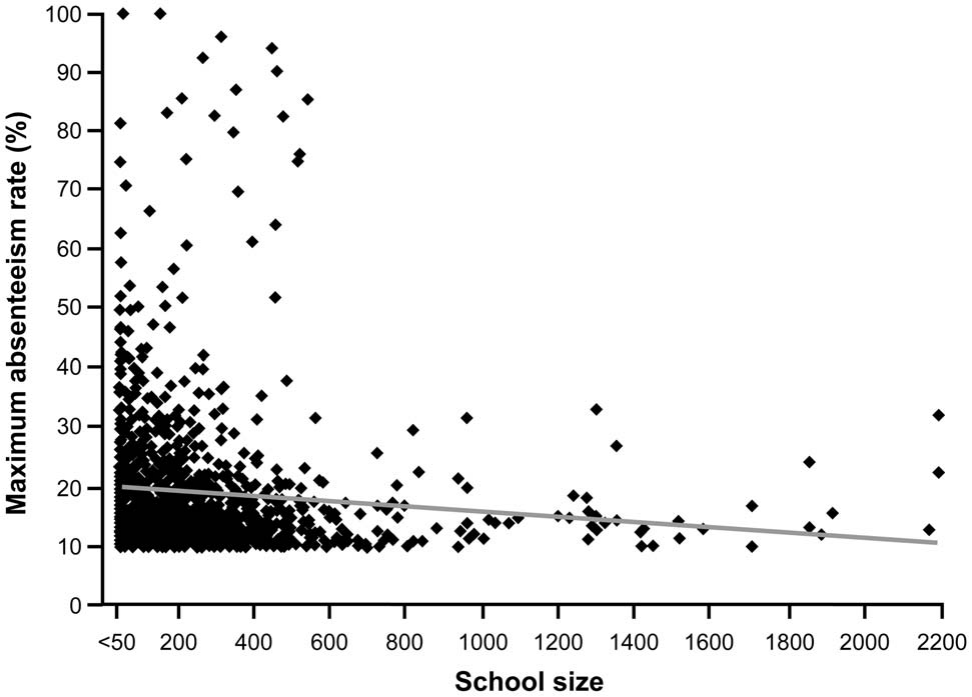
Distribution of the maximal absenteeism rate by school size (all schools) and regression line.

The maximum absenteeism rate was reported the first day by 55% of schools, the second day by 20% and the third by 11% (Table 2). In 71% of schools, the maximum reported absenteeism rate was between 10% and 19% (Table 2). An absenteeism rate ≥20% was reported more often by elementary schools than high schools (29% and 22%, p = 0.01). A maximum absenteeism rate ≥50% was reported by 3% (36/1223) schools including two (with 56 and 154 students) that reported a rate of 100%. The frequency of absenteeism rate ≥20% decreased with school student number: 43% of schools with <100 students, 31% in those with 100 to 299 students, 26% of those with 300 to 499 students and 19% of schools with more than 500 students (p≤0.001). The maximum absenteeism rate showed greater variability in smaller than larger schools. The linear regression analysis showed a significant change in rate by school size (< = −0.004, p = 0.003) but no significant change by education levels (elementary vs high school) (p = 0.82) (Figure 5). There was no relationship between school size and the timing of the first reporting.

**Table 2:** Distribution of the maximum absenteeism rate reported by schools and interval between first reporting of ≥10% absenteeism and the maximum rate in the school.

	Number of schools	Proportion of schools
Maximum absenteeism rate		
10–14%	575	47%
15–19%	291	24%
20–24%	162	13%
25–29%	68	6%
30–34%	41	3.5%
35–39%	27	2%
40–44%	18	1%
≥45%	41	3.5%
Total	1223	100%
Interval in days between first reporting and maximal rate		
0	674	55%
1	245	20%
2	132	11%
3	79	6%
4+	93	8%
Total	1223	100%

### Comparison of school absenteeism surveillance with other indicators

**Figure 6:**
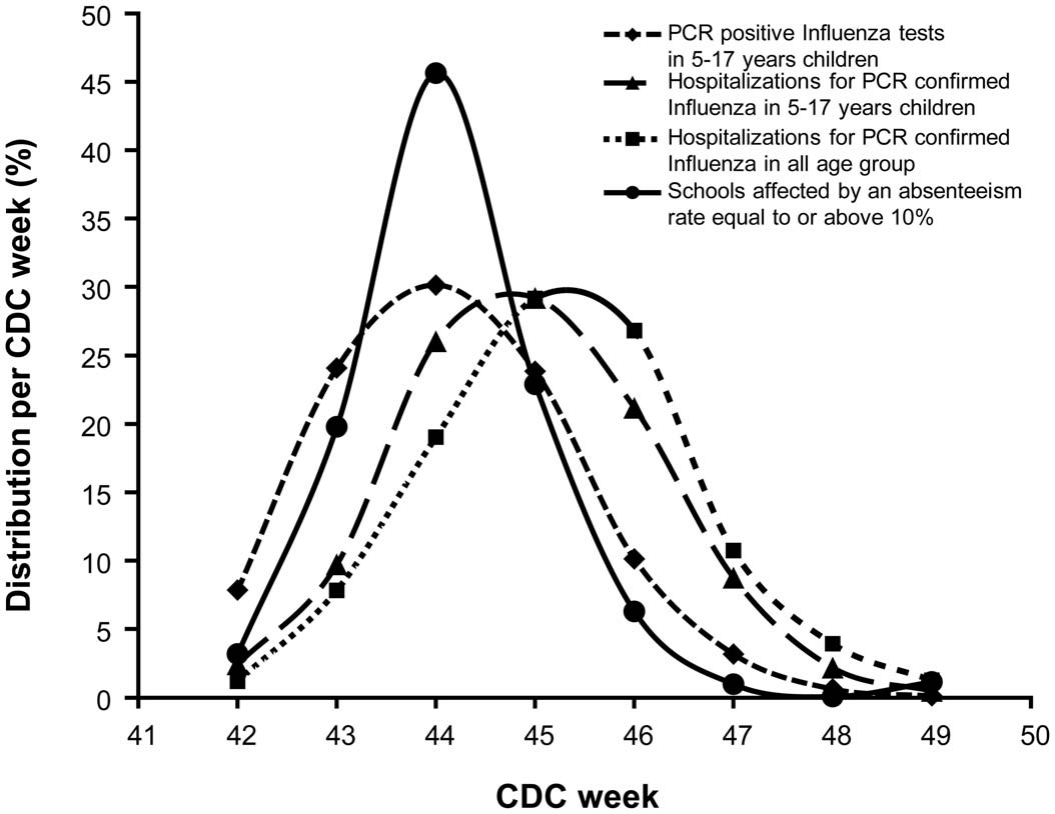
Results from three surveillance indicators: weekly distribution of the number of PCR positive influenza tests in children 5–17 years old (by week specimen found positive), hospitalizations for PCR confirmed Influenza in children 5–17 years old (week of admission) and of schools affected by an absenteeism rate ≥10% (week of school report).

Increase in the number of schools with absenteeism rate ≥10% generally coincided with the increase in PCR-positive influenza tests and hospitalizations. The peak in school absenteeism reports preceded by just one week the peak in hospitalizations among children 5–17 years old(Figure 6). The same pattern was observed when comparing the peak of affected schools with trends in PCR-positive influenza tests and hospitalizations for the general population. Specifically, the peak of affected schools and of positive PCR influenza tests occurred on CDC week 44, whereas the peak of hospitalizations for PCR-confirmed influenza in 5–17 year old children and in all age groups occurred on week 45(Figure 6). Compared to the school absenteeism curve, the Spearman correlation coefficient for hospitalizations in school age children and positive PCR influenza tests was 0.83 (p = 0.01) and 0.90 (p = 0.02) respectively.

## Discussion

During the second wave of the 2009 pandemic in Quebec, about one-third of schools exceeded a 10% threshold for ILI absenteeism at least once during the study period. Smaller schools were more likely to reach that threshold and elementary schools were also more likely than high schools. There were some differences in the number of school outbreaks by region. While initial school outbreak reports provided some advance indication of community activity, the peak in school absenteeism reports mirrored that of other surveillance indicators implemented during the pandemic.

The 10% threshold for school outbreak reporting was chosen based on national guidelines but may warrant revision depending upon stated surveillance objectives. School absenteeism surveillance may serve various objectives including those related to a given affected school or to the broader community. At the individual school level, outbreak reporting may be considered in order to implement interventions to influence transmission and protect vulnerable children (eg. by reinforcing vaccine or antiviral recommendations) within that school. At the community level, initial school outbreak reports may be interpreted as the harbinger of influenza upswing in the population generally and as the trigger to reinforce broader public health messaging.

For the first objective, our study suggests that a 10% absenteeism threshold lacks the sensitivity needed for timely intervention within a given school. In this study, 55% of schools experienced their peak of influenza transmission on the day that absenteeism >10% was first reported and in another 31% the peak rapidly followed within the next two days. As influenza transmission is generally explosive, the mean number of days between 10% absenteeism in a single classroom and the peak of transmission in the entire school is short relative to the delay between obtaining the data and implementing the intervention. In that regard, school outbreak surveillance based on a 10% absenteeism threshold does not appear to be an effective or efficient approach for mitigating within-school impact.

The second objective of using school-based surveillance as an early warning signal for general community upswing has been more commonly promoted as rationale for such a system. Explosive school outbreaks are more dramatic than cases distributed throughout the community and in that sense may be more readily discernible as a realtime indicator. As our school surveillance began on week 42, we could not assess its capacity to detect the very first affected schools in the community. We found the peak incidence of schools with absenteeism ≥10% preceded the peak of pandemic hospitalizations during the second wave by just one week. The earliest school outbreak reports were received about two weeks in advance. This result is consistent with those seen elsewhere [1], [3], [15] and suggests a narrow opportunity for public health response. Whether two weeks constitutes sufficient advance warning will depend upon the capacity for public health to mobilize and communicate rapidly across the region. Hospitalizations during this pandemic included a greater proportion of pediatric cases than during typical seasonal influenza for which the elderly are far more affected. Whether a greater, more practical and relevant lag may exist between initial school outbreak reports and the peak in elderly or other hospitalizations during seasonal (versus pandemic) influenza warrants specific evaluation. Furthermore, during seasonal influenza laboratory testing is less routinely conducted in the general population and in hospitalized patients than was the case in Quebec during the pandemic. Because of the weakening of these other components of seasonal influenza surveillance, the added value of school-based ILI outbreak surveillance may also be greater during typical seasonal influenza. A 10% absenteeism threshold may be insufficient to detect the *very first* school outbreak in an area but given contribution across all schools such a fine level of detection may not be necessary: detection of the initial few school reports may suffice for population purposes.

That 35% of schools reached the 10% threshold during the fall pandemic wave – before the peak and before vaccine became available to school-aged children – also warrants consideration. Given broad susceptibility and high incidence of pandemic influenza in the population during the second wave, it is unlikely that the remaining 65% of schools were exempt. This suggests that the applied threshold identified only the most severely affected schools which may be relevant to surveillance objectives. The threshold selected should also take into account the positive predictive value of ILI-related absenteeism. In the context of this second wave of the pandemic where few other viruses co-circulated with A (H1N1) influenza and the proportion of specimens that tested positive for influenza in Quebec reached 45%–50%, the positive predictive value was high. It is likely lower during regular influenza seasons with contribution from non-influenza viruses and a maximum proportion of specimens that test positive for influenza ranging between 25% and 35% [18]. Few other respiratory viruses, however, demonstrate the kind of explosive spread and severe illness that influenza manifests through absenteeism.

Our study suggests that the preferred school outbreak threshold should also take into account the school size. The percentage of schools with 100–299 students that reached the assigned threshold was twice that of schools with 500 or more students (22% vs 44%). The maximum absenteeism rate and the duration of reporting varied more in smaller than larger schools but the regression analysis showed no significant effect of the size of the school or the education level. That some small schools had absenteeism rates reaching 50% or more while this did not happen in larger schools should not be interpreted as an indication that smaller school have greater risks. This reflects the greater variability expected when sample size (school size) is small [19]. Transmission is likely to be higher within rather than between classes and because larger schools typically have more classes, the likelihood of reaching the higher threshold overall will be lower. The optimal school-based surveillance threshold warrants further reflection incorporating these nuanced considerations. A threshold of 10% in a single day in a classroom (e.g. 3 students in a group of 25–30) is a more sensitive indicator than applying the 10% threshold to an entire school regardless of its size. However, a classroom threshold of 10% absenteeism for ILI has lower positive predictive value for indicating influenza activity than the school threshold.

Outside Canada different thresholds have been applied to school-based surveillance. In a study conducted in Japan [7], a daily influenza-related absenteeism rate of 10% was chosen as threshold, based on the 95^th^ percentile of daily absentee rate (10.7%) in a certain number of elementary schools. A threshold of 7.5% absenteeism rate has also been used in a study conducted in Boulder County in the US [1]. In the Boulder County school-based surveillance system, each school was required to telephone a report each Friday whenever the weekly average rate exceeded 7.5% of the current school census. Epidemic curves based on these school data were correlated with those of sentinel surveillance systems [1]. However the rationale for this threshold is unclear and the study did not differentiate large and small schools, making comparisons difficult. In Japan, various thresholds of absenteeism have been suggested in considering school closure [7]. The analysis took into account the sensitivity and the specificity of the indicator using the Youden index calculated as (sensitivity+specificity) -1. This test suggested a school absenteeism threshold of 5% during a single day or ≥4% for two days, or ≥3% for three days.

Additional returns on the investment in a school-based surveillance system may include quantifiable indicators of impact and epidemic intensity that could be compared retrospectively (rather than realtime) across seasons or epidemics. This includes estimation of schools currently affected at a recognized stage of the epidemic evolution (prevalence), or all schools affected since the beginning of the season (cumulative incidence of schools affected), or as an estimate of the proportion of the total enrolment of children infected at one point or by the end of season (attack rate in children). Computerized daily absenteeism data in each school would facilitate rapid tracking to meet these objectives. The optimal parameters for these and other objectives of school-based monitoring would also require evaluation if they are to be considered a component of routine surveillance.

This study has limitations. Quebec experienced a substantial first wave of the pandemic during the spring period of the prior school year (April–May 2009), especially in the Montreal area which accounted for 50% of all first wave cases. While school absenteeism surveillance was not in place during the first wave to compare with the second wave, this high transmission in Montreal during the first wave may explain its lower absenteeism profile compared to adjacent areas during the second wave. Our school surveillance was first introduced during the second wave and there were no baseline data on absenteeism in schools for comparison. Data collection was delayed until two weeks after the beginning of the 2^nd^ wave so that the true utility of school-based reporting as an early warning system for community upswing may have been missed. Instructions were also not strictly followed by schools: while schools were requested to report for 7days after the last occurrence of the 10% threshold, 35% of schools reported absenteeism ≤3days. There was no direct quality assessment of how data were compiled at the school level. Despite that, it is reassuring to see that the trends were similar to those observed with other surveillance systems. School-based absenteeism reporting, as for other surveillance processes, may therefore be considered useful for tracking general trends (timeliness and intensity) but should not be interpreted literally as an absolute measure of disease burden. Our use of the number of influenza PCR positive tests by week, rather than the proportion positive per week, may similarly be considered reliable for trend tracking as both indicators followed almost identical temporal trends [20]. Finally, it is important to reinforce that pandemic activity may be very different from that of seasonal influenza and it is difficult to compare surveillance indicators on that basis. For instance, we compared school-based surveillance with surveillance of hospitalizations among children 5–17 years of age during the pandemic. While the latter may be useful during the dramatic activity observed during a pandemic, hospitalization for influenza is rare among school-age children during regular winter seasons and as such, school outbreak surveillance may yield greater added value during seasonal compared to pandemic influenza activity.

During a pandemic, school outbreak surveillance based on a 10% absenteeism threshold appears insufficient to trigger timely intervention within a given affected school. However, school surveillance appears well-correlated and slightly anticipatory compared to other population indicators and may also perform better during seasonal influenza. As such, school absenteeism may be considered an adjunct surveillance indicator whose overall utility will depend upon specified objectives and other existing capacity for monitoring and response. The optimal threshold for school outbreak reporting should be aligned with objectives and balance timely and comprehensive data collection with practicality, simplicity, feasibility and sustainability. In that regard, school outbreak surveillance warrants further evaluation.
